# Nurses’ knowledge, attitude, and practice of low-flow oxygen therapy and humidification

**DOI:** 10.3389/fmed.2024.1460079

**Published:** 2024-11-18

**Authors:** Naiwang Tang, Haiying Li, Jiayi Zhang, Hua Ling, Linlin Shi, Huili Zhang, Qi Guo, Ronghuan Yu

**Affiliations:** ^1^Department of Respiratory and Critical Care Medicine, Shanghai Xuhui Central Hospital, Shanghai, China; ^2^Nursing Department, Shanghai Xuhui Central Hospital, Shanghai, China; ^3^Department of Critical Care Medicine, Shanghai Xuhui Central Hospital, Shanghai, China; ^4^Department of Infectious Disease, Shanghai Xuhui Central Hospital, Shanghai, China

**Keywords:** knowledge, attitude, practice, low-flow oxygen therapy, humidification, nurses

## Abstract

**Objective:**

Nurses are key in administering oxygen therapy and managing its potential adverse effects in medical settings. This study aimed to investigate the knowledge, attitudes, and practices (KAP) regarding low-flow oxygen therapy and humidification among nurses.

**Methods:**

This cross-sectional study was conducted from January 2024 to March 2024 at Shanghai Xuhui Central Hospital. Demographic data and KAP scores were collected through questionnaires.

**Result:**

A total of 243 valid questionnaires were collected. Among them, 228 (93.8%) were female, and 93 (38.3%) had been working for more than 10 years. The mean scores for knowledge, attitudes, and practices were 11.11 ± 4.30 (Ranging 0–18), 29.14 ± 3.41 (Ranging 7–35), and 28.07 ± 4.73 (Ranging 7–35), respectively. Multivariate logistic regression confirmed that knowledge (OR = 1.302, 95% CI: [1.167–1.453], *p* < 0.001) and attitudes (OR = 1.196, 95% CI: [1.080–1.325], *p* < 0.001) were independently associated with proactive practices. Structural equation modeling (SEM) corroborated the direct influences of training (*β* = 3.210, *p* < 0.001) and clinical experience (*β* = 2.044, *p* = 0.002) on knowledge, with knowledge (*β* = 0.379, *p* < 0.001) and gender (*β* = −1.642, *p* = 0.037) directly impacting attitudes. Additionally, knowledge (β = 0.395, *p* < 0.001), attitudes (*β* = 0.340, *p* < 0.001), and equipment utilization (*β* = 1.098, *p* < 0.001) directly influenced practices.

**Conclusion:**

Nurses demonstrated inadequate knowledge, positive attitudes, and inactive practices toward low-flow oxygen therapy and humidification. Enhanced training and increased clinical experience are recommended to improve nurses’ knowledge and practice in this area.

## Introduction

Oxygen therapy is the primary treatment for hypoxemia, aiming to enhance arterial oxygen pressure. This method is particularly vital in the Department of Respiratory Medicine, where hypoxemia frequently occurs due to inadequate gas exchange in the lungs, caused by factors such as hypoventilation, ventilation/perfusion (V/Q) mismatch, intrapulmonary right-to-left shunting, diffusion impairment, or combinations of these (1). If administered correctly, oxygen therapy can greatly improve treatment outcomes and even save lives. However, its improper use may lead to severe complications (2).

Oxygen is delivered through various systems, categorized into low-flow and high-flow. Low-flow systems provide oxygen at rates below the patient’s inspiratory flow rate, around 30 L/min, whereas high-flow systems exceed the patient’s inspiratory flow in the hope of ensuring adequate oxygenation (3). The devices used in these systems range from face masks and nasal cannulas to more specialized options like Venturi masks, partial and non-re-breather masks, and trans-tracheal catheters (4, 5). Among these, the high-flow nasal cannula (HFNC) has gained prominence for delivering a heated and humidified high-flow air-oxygen mixture. Studies have demonstrated that HFNC outperforms conventional oxygen therapy methods in effectiveness (6–8). Conversely, the routine humidification of oxygen in low-flow systems is not always justifiable. Non-humidified oxygen is particularly advantageous as it reduces bacterial contamination in humidifier bottles, decreases the risk of respiratory infections, and maintains effective oxygen administration times without significant adverse effects on patient comfort or oxygen saturation levels (9).

The Knowledge-Attitude-Practice (KAP) model is crucial in understanding and shaping health behaviors, serving as a foundational component of health literacy. This model posits that knowledge positively influences attitudes, which in turn affect practices (10). It is commonly utilized in conjunction with the KAP questionnaire to thoroughly assess the knowledge, attitudes, and practices of healthcare professionals, as well as to evaluate the demand for and acceptance of relevant interventions within the healthcare sector (11). Nurses play a critical role in administering oxygen therapy and addressing its potential adverse effects in medical centers (12). However, research indicates a substantial knowledge gap among nurses regarding the correct use of oxygen therapy, which has been observed in various international studies (13–15).

Despite the broad application of the KAP model in healthcare research, there is a notable absence of studies specifically exploring low-flow oxygen therapy and humidification among nurses. Addressing this gap is essential for improving the standard of care and minimizing risks associated with oxygen therapy. Consequently, this study aims to investigate the knowledge, attitudes, and practices concerning low-flow oxygen therapy and humidification among nurses, to identify and address these specific educational needs.

## Materials and methods

### Study design and subjects

This cross-sectional study was conducted from January 2024 to March 2024 at Shanghai Xuhui Central Hospital, focusing on the nursing staff. This study was approved by Shanghai Xuhui Central Hospital Ethics Committee [(2023) Court Review No. (046)], and all participants provided written informed consent.

Nursing staff employed at hospital from January 2024 to March 2024 were included in the study. Exclusion criteria encompassed those who did not submit or selected “No” on the informed consent form, those who had not formally signed a labor contract with hospital, including dispatched personnel and exchange students, and populations deemed unsuitable for inclusion by other researchers.

The electronic questionnaire was created using the Questionnaire Star platform, with a QR code provided to participants. Respondents could access and complete the survey by scanning the QR code via WeChat or by using the provided link. To ensure data integrity and comprehensiveness, submissions were restricted to one per IP address, and all survey items were mandatory. Anonymity was guaranteed for all participants throughout the survey.

### Questionnaire introduction

The questionnaire was designed based on established guidelines and literature (9, 16–21) and refined with input from four experts (one respiratory expert and three nursing experts). After incorporating their feedback, a preliminary version of the questionnaire was distributed on a small scale (51 copies), yielding a reliability score of 0.868.

Following ethical approval, the first version of the questionnaire was distributed on a small scale across various departments, including internal medicine and surgery, collecting 2–4 questionnaires per department and resulting in 40 responses. After excluding one respondent who disagreed with the study, three who completed the questionnaire in less than 90 s (22), and eleven who answered trap questions incorrectly, 25 valid questionnaires remained. The initial results showed a Cronbach’s *α* coefficient of −0.244 for the “knowledge” section, necessitating a redesign. The knowledge section was modified to a popular science format, assessing respondents’ understanding of key points rather than selecting correct answers. Additional validity testing yielded a Kaiser-Meyer-Olkin (KMO) value of 0.897, indicating strong validity of the revised instrument.

The revised questionnaire underwent another round of ethical review and was distributed on a second small scale, receiving 51 responses. After excluding five responses with completion times less than 90 s and nineteen with incorrectly answered trap questions, 27 valid questionnaires remained. This version achieved an overall Cronbach’s *α* coefficient of 0.868, with coefficients of 0.787, 0.864, and 0.817 for the knowledge, attitude, and practice sections, respectively. Finally, the questionnaire was distributed to all nursing staff, resulting in 399 responses. Exclusions included four responses with completion times less than 60 s, one respondent who disagreed with the study, and 151 respondents who answered trap questions incorrectly, leaving 243 valid responses.

The final questionnaire, written in Chinese, included four dimensions with a total of 33 items. These dimensions were: basic information (9 items), knowledge (10 items, including 10 trap questions to identify invalid responses), attitude (7 items), and practice (7 items). During statistical analysis, scores were assigned based on response options. For the knowledge dimension, scores were 2 points for “very familiar,” 1 point for “heard of,” and 0 points for “unclear,” with a total possible score ranging from 0 to 18. For the attitude dimension, responses ranged from “strongly agree” to “strongly disagree,” scoring from 5 to 1, with a total possible score from 7 to 35. For the practice dimension, responses ranged from “always” to “never,” also scoring from 5 to 1, with a total possible score from 7 to 35.

### Sample size calculation

The sample size criterion requires that the minimum sample size be 5–20 times the number of items (23). With 33 items in the questionnaire, the minimum sample size was calculated to be 165. Allowing for a 20% non-response rate, the required sample size was adjusted to 207. This study successfully enrolled a total of 399 participants.

### Statistical analysis

Data analysis was conducted using SPSS 27.0 and Amos 26.0 (IBM, Armonk, NY, USA). Questionnaire reliability was assessed with Cronbach’s *α*. Descriptive analysis of demographic information and KAP scores presented continuous data as Mean ± SD and categorical data as frequency (percentage). Differences in knowledge (K), attitude (A), and practice (P) scores across demographic groups were analyzed. Normally distributed continuous variables were compared using the t-test, while non-normally distributed variables were analyzed using the Mann–Whitney U test or Kruskal-Wallis H test. Spearman correlation analysis evaluated the relationships between K, A, and P, with coefficients ranging from −1 to +1. Logistic regression explored the impact of demographic information, knowledge, and attitude on practice, classifying practice scores at 70% (24). Path analysis examined the relationships between baseline information and KAP dimensions. Statistical significance was set at a *p*-value of less than 0.05.

## Result

Initially, a total of 399 questionnaires were collected in this study, and data from 4 respondents who completed the questionnaire in less than 60 s, 1 who declined participation, and 151 who incorrectly answered a control question were excluded, resulting in 243 valid responses. The Cronbach’s *α* coefficient calculated from these 243 responses was 0.905, indicating strong internal consistency. Of the participants, 228 (93.8%) were female, with mean age of 32.21 ± 9.74 years, 134 (55.10%) had a Bachelor’s degree or higher, 93 (38.30%) were nurse practitioners, 93 (38.30%) had been working for more than 10 years, 153 (63.00%) had received specialized education or training in low-flow oxygen therapy, 199 (81.90%) had dealt with patients requiring low-flow oxygen therapy, 185 (76.10%) have used humidification equipment to assist low-flow oxygen therapy in their daily practice. The mean knowledge, attitude, and practice scores were 11.11 ± 4.30, 29.14 ± 3.41, and 28.07 ± 4.73, respectively. The knowledge score varied from participants with different department (*p* = 0.005), education or training status (*p* = 0.001), whether dealt with patients requiring low-flow oxygen therapy (*p* = 0.001), and whether used humidification equipment (*p* = 0.001). As for the attitude score, there were difference among those with different gender (*p* = 0.023), education or training status (*p* = 0.001), and whether dealt with patients requiring low-flow oxygen therapy (*p* = 0.044). The difference of practice score were found among those with different education or training status (*p* = 0.001), whether dealt with patients requiring low-flow oxygen therapy (*p* = 0.001), and whether used humidification equipment (*p* = 0.001) (as shown in Table 1).

**Table 1:** Baseline.

	*N* (%)	Knowledge, mean ± SD	*p*	Attitude, mean ± SD	*p*	Practice, mean ± SD	*p*
*N* = 243							
Total score		11.11 ± 4.30		29.14 ± 3.41		28.07 ± 4.73	
Gender			0.112		0.023		0.147
Male	15 (6.20%)	12.67 ± 3.46		31.27 ± 2.58		29.53 ± 4.34	
Female	228 (93.8%)	11.01 ± 4.35		29.00 ± 3.42		27.98 ± 4.75	
Age	32.21 ± 9.74						
Education			0.219		0.069		0.108
College	109 (44.90%)	10.72 ± 4.35		28.66 ± 3.38		27.62 ± 4.68	
Bachelor’s degree and above	134 (55.10%)	11.43 ± 4.27		29.52 ± 3.40		28.44 ± 4.76	
Department			0.005		0.111		0.189
Department of Respiratory and Critical Care Medicine	21 (8.60%)	11.81 ± 3.97		30.14 ± 3.51		27.67 ± 4.45	
Cardiology	24 (9.90%)	10.67 ± 4.04		28.83 ± 3.47		28.38 ± 4.07	
Emergency department	24 (9.90%)	11.71 ± 4.16		29.08 ± 3.46		28.88 ± 4.26	
Department of Intensive Care Medicine (ICU)	26 (10.70%)	9.50 ± 2.64		28.69 ± 3.50		27.96 ± 4.36	
Surgical	23 (9.50%)	8.70 ± 4.61		27.48 ± 3.04		25.35 ± 5.74	
Other departments	125 (51.40%)	11.74 ± 4.48		29.43 ± 3.36		28.46 ± 4.76	
Professional title			0.741		0.247		0.883
Nurse	85 (35.00%)	10.84 ± 4.55		29.40 ± 3.37		28.00 ± 4.83	
Nurse practitioner	93 (38.30%)	11.29 ± 4.20		28.68 ± 3.54		28.11 ± 4.59	
Nurse practitioner in charge and above	65 (26.70%)	11.22 ± 4.19		29.45 ± 3.25		28.12 ± 4.86	
Years of work experience			0.546		0.846		0.732
Less than 1 year	23 (9.50%)	10.30 ± 3.78		29.65 ± 3.16		27.17 ± 4.07	
1–3 years	43 (17.70%)	10.47 ± 4.55		29.35 ± 3.54		27.77 ± 4.99	
3–5 years	40 (16.50%)	11.15 ± 4.62		28.68 ± 3.34		28.48 ± 4.34	
5–10 years	44 (18.10%)	11.75 ± 4.55		29.25 ± 3.84		28.34 ± 4.20	
More than 10 years	93 (38.30%)	11.29 ± 4.08		29.05 ± 3.27		28.14 ± 5.18	
Have you received any special education or training in low-flow oxygen therapy?			0.001		0.001		0.001
Yes	153 (63.00%)	12.52 ± 4.01		29.92 ± 3.28		29.30 ± 4.31	
No	90 (37.00%)	8.72 ± 3.73		27.81 ± 3.23		25.99 ± 4.70	
Have you ever dealt with patients requiring low-flow oxygen therapy?			0.001		0.044		0.001
Yes	199 (81.90%)	11.78 ± 4.21		29.36 ± 3.41		28.97 ± 4.17	
No	44 (18.10%)	8.09 ± 3.38		28.14 ± 3.25		24.02 ± 5.02	
Whether humidification equipment is used in daily practice to assist with low-flow oxygen therapy			0.001		0.124		0.001
Yes	185 (76.10%)	11.86 ± 4.19		29.35 ± 3.36		29.16 ± 4.14	
No	14 (5.80%)	10.64 ± 4.41		29.50 ± 4.26		26.43 ± 3.86	
Not processed	44 (18.10%)	8.09 ± 3.38		28.14 ± 3.25		24.02 ± 5.02	

The distribution of knowledge dimension revealed that the question with the highest number of participants choosing the “Very familiar” option was “When using a humidifier, the quality of the water is crucial. It is recommended to use pure distilled or deionized water to prevent the introduction of bacteria or contaminants into the respiratory tract.” (K7), with 51.90%. The question with the highest number of participants choosing the “Moderately familiar” option was “The oxygen supply system is divided into a low-flow system and a high-flow system. The low-flow system is a device that provides an oxygen flow rate of less than 30 liters/min.” (K1), with 60.90%. The question with the highest number of participants choosing the “Not familiar” option was “The upper respiratory tract (nasal cavity, throat) can provide 75% of heat and humidity. The American Respiratory Therapy Association proposes that humidification of oxygen is not required when the oxygen flow is less than 4 liters/min.” (K5), with 28.00% (as shown in Table 2).

**Table 2:** Knowledge dimension.

	*N* (%)		
	Very familiar	Moderately familiar	Not familiar
1. The oxygen supply system is divided into a low-flow system and a high-flow system. The low-flow system is a device that provides an oxygen flow rate of less than 30 liters/min.	67 (27.60%)	148 (60.90%)	28 (11.50%)
2. Under normal circumstances, when the oxygen flow rate is less than 6 liters/min, only a nasal cannula needs to be used; when the oxygen flow rate exceeds 6 liters/min, a mask needs to be used.	125 (51.40%)	113 (46.50%)	5 (2.10%)
3. Studies have shown that oxygen is not easily soluble in water, so the oxygen humidification bottle used in low-flow oxygen therapy cannot effectively humidify oxygen.	57 (23.50%)	125 (51.40%)	61 (25.10%)
4. The use of a heated humidifier during low-flow oxygen therapy can help promote the vibration of airway mucus and cilia, hydrate and dilute mucus and sputum, and improve lung function.	103 (42.40%)	121 (49.80%)	19 (7.80%)
5. The upper respiratory tract (nasal cavity, throat) can provide 75% of heat and humidity. The American Respiratory Therapy Association proposes that humidification of oxygen is not required when the oxygen flow is less than 4 liters/min.	58 (23.90%)	117 (48.10%)	68 (28.00%)
6. When the nasal cannula supplies oxygen at 4 liters/min, the oxygen concentration inhaled by the patient when inhaling quickly is lower than when inhaling calmly.	67 (27.60%)	121 (49.80%)	55 (22.60%)
7. When using a humidifier, the quality of the water is crucial. It is recommended to use pure distilled or deionized water to prevent the introduction of bacteria or contaminants into the respiratory tract.	126 (51.90%)	109 (44.90%)	8 (3.30%)
8. When using ordinary oxygen masks for low-flow oxygen therapy, the recommended oxygen flow is 6–15 liters/min.	102 (42.00%)	128 (52.70%)	13 (5.30%)
9. A hypoxemic patient with an oxygen saturation of 87% is accompanied by hypercapnia (increased CO2 partial pressure in blood gas analysis). Through low-flow oxygen therapy, the target oxygen saturation that should be achieved is 88–92%.	86 (35.40%)	136 (56.00%)	21 (8.60%)

Responses on attitudes showed that patient’s attitudes tended to be positive, with 61.30% strongly agreeing that patients should be properly educated and instructed during low-flow oxygen therapy (A1), 59.30% strongly agreeing that nurses should receive regular training and continuing education on low-flow oxygen therapy (A2), as well as 53.50% strongly agreeing that humidification helps to reduce patients’ discomfort during low-flow oxygen therapy (A4). However, when it comes to whether the replacement of humidifying bottles and canisters would increase their workload (A6), 31.70% strongly agree, 32.10% agree, 16.90% were neutral, and 13.20% were against (as shown in Table 3).

**Table 3:** Attitude dimension.

	*N* (%)				
	Strongly agree	Agree	Neutral	Disagree	Strongly disagree
1. I believe patients should receive appropriate education and guidance while receiving low-flow oxygen therapy. P	149 (61.30%)	86 (35.40%)	8 (3.30%)	0	0
2. I believe that nurses should receive regular training and continuing education on low-flow oxygen therapy. P	144 (59.30%)	88 (36.20%)	11 (4.50%)	0	0
3. I think providing humidification bottles and humidification tanks for low-flow oxygen therapy is an economical and effective care method. P	121 (49.80%)	96 (39.50%)	22 (9.10%)	2 (0.80%)	2 (0.80%)
4. I think humidification helps reduce patient discomfort during low-flow oxygen therapy. P	130 (53.50%)	97 (39.90%)	15 (6.20%)	1 (0.40%)	0
5. I think humidification is very important to reduce complications in patients receiving low-flow oxygen therapy. P	121 (49.80%)	103 (42.40%)	17 (7.00%)	2 (0.80%)	0
6. I think replacing humidification bottles and humidification tanks will increase my workload. N	77 (31.70%)	78 (32.10%)	41 (16.90%)	32 (13.20%)	15 (6.20%)
7. I think nurses should actively participate in the research and improvement of low-flow oxygen therapy humidification. P	132 (54.30%)	93 (38.30%)	17 (7.00%)	1 (0.40%)	0

Turning to related practices, 53.50% always checked the operational status of the oxygen equipment (P4), 47.30% always monitored the patient’s oxygen saturation during low-flow oxygen therapy (P2), and 46.10% always collaborated with the patient’s treating physician to ensure that low-flow oxygen therapy was performed effectively (P3). Strikingly, 18.10% occasionally and 26.70% never made their own decisions about the oxygen flow rate to be used for the patient’s oxygen therapy during nursing care (P6) (as shown in Table 4).

**Table 4:** Practice dimension.

	*N* (%)				
	Always	Often	Sometimes	Occasionally	Never
1. During the nursing process, you always adjust low-flow oxygen therapy devices such as nasal cannulas and ordinary oxygen masks according to the needs and conditions of the patient. P	106 (43.60%)	99 (40.70%)	34 (14.00%)	4 (1.60%)	0
2. You always monitor the patient’s oxygen saturation during low-flow oxygen therapy. P	115 (47.30%)	92 (37.90%)	26 (10.70%)	9 (3.70%)	1 (0.40%)
3. You always work with the patient’s treating physician to ensure the effective implementation of low-flow oxygen therapy. P	112 (46.10%)	97 (39.90%)	23 (9.50%)	10 (4.10%)	1 (0.40%)
4. You always check the operation of your oxygen equipment to ensure that your humidification equipment is correct and working properly. P	130 (53.50%)	82 (33.70%)	24 (9.90%)	6 (2.50%)	1 (0.40%)
5. You always provide patients with information about the benefits and complications of low-flow oxygen therapy. P	91 (37.40%)	87 (35.80%)	49 (20.20%)	15 (6.20%)	1 (0.40%)
6. You decide the oxygen flow rate used for the patient’s oxygen therapy during care. P	44 (18.10%)	46 (18.90%)	44 (18.10%)	44 (18.10%)	65 (26.70%)
7. When you perform humidification, always educate patients on the correct use and maintenance of humidifiers. P	89 (36.6%)	90 (37.00%)	45 (18.50%)	16 (6.60%)	3 (1.20%)

In the correlation analysis, significant positive correlations were found between knowledge and attitude (*r* = 0.465, *p* < 0.001), knowledge and practice (r = 0.592, p < 0.001), as well as attitude and practice (*r* = 0.480, *p* < 0.001), respectively (as shown in Table 5).

**Table 5:** Correlation analysis.

	Knowledge	Attitude	Practice
Knowledge	1		
Attitude	0.465(*p* < 0.001)	1	
Practice	0.592(*p* < 0.001)	0.480(*p* < 0.001)	1

Multivariate logistic regression showed that knowledge score (OR = 1.302, 95% CI: [1.167–1.453], *p* < 0.001) and attitude score (OR = 1.196, 95% CI: [1.080–1.325], *p* < 0.001) were independently associated with proactive practice (as shown in Table 6).

**Table 6:** Multivariate logistic regression analysis for practice.

Practice (a total score of more than 80% is active practice)	Univariate logistic regression		Multivariate logistic regression	
	OR (95%CI)	*p*	OR (95%CI)	*p*
Knowledge score	1.368(1.247–1.502)	<0.001	1.302(1.167–1.453)	<0.001
Attitude score	1.324(1.208–1.451)	<0.001	1.196(1.080–1.325)	<0.001
Gender				
Male	2.561(0.703–9.330)	0.154		
Female	ref			
Age	1.007(0.979–1.035)	0.649		
Education				
College	ref			
Bachelor’s degree and above	0.667(0.396–1.124)	0.128		
Department				
Department of Respiratory and Critical Care Medicine	0.582(0.226–1.498)	0.262	0.318(0.100–1.010)	0.052
Cardiology	0.611(0.249–1.499)	0.282	0.696(0.237–2.038)	0.508
Emergency department	0.611(0.249–1.499)	0.282	0.465(0.154–1.406)	0.175
Department of Intensive Care Medicine (ICU)	0.374(0.158–0.885)	0.025	0.497(0.189–1.311)	0.158
Surgical	0.476(0.193–1.175)	0.107	1.202(0.399–3.621)	0.743
Other departments	ref		ref	
Professional title				
Nurse	ref			
Nurse practitioner in charge and above	0.756(0.388–1.472)	0.410		
Years of work experience				
Less than 1 year	ref			
1–3 years	0.715(0.283–1.807)	0.478		
3–5 years	0.764(0.364–1.601)	0.476		
5–10 years	0.744(0.349–1.587)	0.444		
10 years and above	1.179(0.549–2.529)	0.673		
Have you received any special education or training in low-flow oxygen therapy?				
Yes	2.432(1.419–4.168)	0.001	0.815(0.397–1.671)	0.577
No	ref		ref	
Have you ever dealt with patients requiring low-flow oxygen therapy?				
Yes	2.911(1.490–5.686)	0.002	1.794(0.778–4.134)	0.170
No	ref		ref	
Whether humidification equipment is used in daily practice to assist with low-flow oxygen therapy				
Yes	2.136(0.716–6.367)	0.173		
No	Ref			

**Figure 1:**
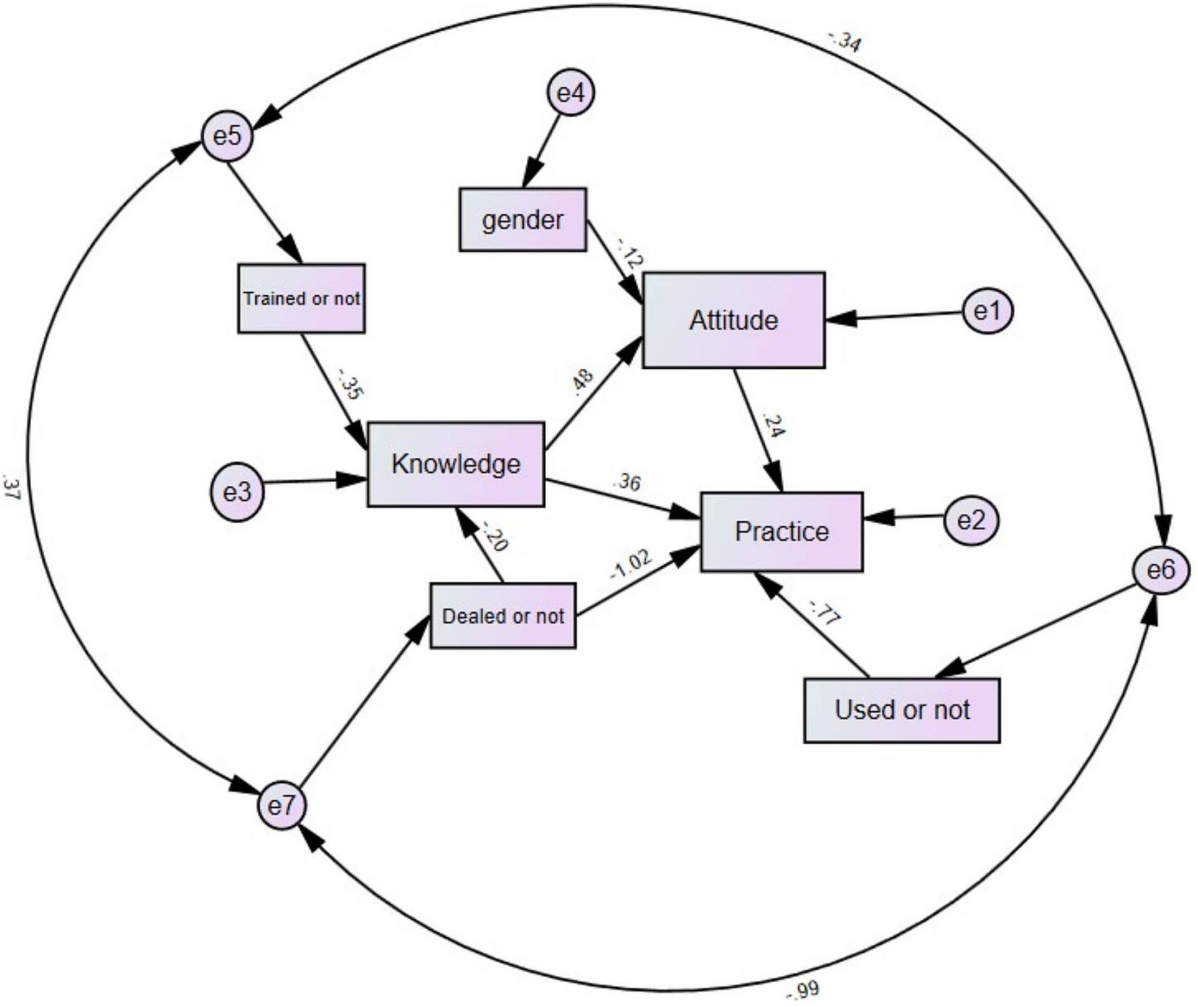
Path analysis results. “Trained or not” refers to the variable in the baseline indicating whether the individual has received specialized education or training on low-flow oxygen therapy. “Dealed or not” refers to the variable in the baseline indicating whether the individual has dealt with patients requiring low-flow oxygen therapy. “Used or not” refers to the variable in the baseline indicating whether the individual has used humidification devices to assist with low-flow oxygen therapy in daily practice. “Gender” refers to the variable in the baseline indicating the individual’s gender. This model illustrates the relationships between gender, training, experience (“dealed or not”), equipment use (“used or not”), and nurses’ knowledge, attitudes, and practices concerning low-flow oxygen therapy. Arrows represent the hypothesized directions of influence. Standardized path coefficients are shown next to each arrow, indicating the strength of these relationships. Error terms (e1 to e7) are associated with the corresponding latent variables, accounting for unexplained variance.

The fit indices of the SEM model reached the desired range, indicating good model fit results (as shown in Supplementary Table S1), SEM results show that ‘Trained or not’ (*β* = 3.210, *p* < 0.001) and ‘Dealed or not’(*β* = 2.044, *p* = 0.002) directly affected knowledge. Meanwhile, knowledge (*β* = 0.0.379, *p* < 0.001) and gender (*β* = −1.642, *p* = 0.037) directly affected attitude. Further, knowledge (*β* = 0.395, *p* < 0.001), attitude (*β* = 0.340, *p* < 0.001), and ‘Used or not’ (*β* = 1.098, *p* < 0.001) directly affected practice (Figure 1; Supplementary Table S2).

## Discussion

Nurses demonstrated inadequate knowledge, positive attitudes, and inactive practices toward low-flow oxygen therapy and humidification. It is essential to implement comprehensive training programs and continuous professional development initiatives to enhance nurses’ knowledge and practices related to low-flow oxygen therapy.

This study highlights that nurses demonstrated inadequate knowledge, positive attitudes, and inactive practices toward low-flow oxygen therapy and humidification. Significant differences in knowledge, attitudes, and practices were observed based on gender, department, special training, clinical experience, and the use of humidification equipment. For instance, gender differences were significant in attitudes, with male nurses scoring higher than female nurses. This finding could be influenced by varying educational backgrounds or different experiences in clinical settings between genders (25, 26). However, no significant gender differences were observed in knowledge and practice scores. Besides, departmental differences significantly influenced knowledge scores. Nurses from the Department of Respiratory and Critical Care Medicine scored the highest, likely due to their frequent exposure to and training in oxygen therapy. Conversely, nurses from the Surgical department had the lowest scores, which might reflect less requirement on oxygen therapy in their daily responsibilities.

Moreover, special education or training in low-flow oxygen therapy significantly improved scores across all KAP categories. Nurses who received training scored higher in knowledge, attitudes, and practices. This underscores the importance of targeted educational interventions, which have been shown to enhance healthcare providers’ competencies in various clinical areas (27). Experience with patients requiring low-flow oxygen therapy was a critical factor in improving nurses’ KAP scores, highlighting the importance of hands-on experience. Nurses with this clinical exposure demonstrated greater knowledge, more positive attitudes, and better practices compared to those with purely theoretical training. Practical experience not only reinforces theoretical concepts but also equips nurses with essential skills for managing respiratory conditions, such as identifying early signs of hypoxia, adjusting oxygen flow rates appropriately, and monitoring patient outcomes effectively. By bridging the gap between theory and practice, this experience enhances nurses’ ability to manage complex cases and adapt swiftly to changing patient needs, ultimately contributing to better patient care. The experiential learning theory posits that direct patient care experiences are essential for effective learning (28, 29). The use of humidification equipment in daily practice was associated with higher knowledge and practice scores, indicating that regular use of relevant tools can reinforce learning and application. This practical engagement likely enhances familiarity and confidence in using the equipment, leading to better clinical practices.

Multivariate logistic regression and SEM results highlighted the independent and direct effects of knowledge and attitudes on proactive practices. Knowledge scores were strongly associated with better practices, corroborating findings from other studies that link higher knowledge levels to improved clinical practices (30). Attitudes also significantly influenced practices, suggesting that positive attitudes toward low-flow oxygen therapy can drive better implementation. The correlation analyses and SEM results further elucidated the relationships between KAP dimensions. Significant positive correlations between knowledge and attitudes, knowledge and practices, and attitudes and practices suggest that these elements are interdependent. Enhancing one aspect, such as knowledge through training, is likely to positively impact attitudes and practices as well (31).

The knowledge dimension revealed a varied familiarity among participants regarding low-flow oxygen therapy. While many respondents showed moderate to high familiarity with concepts such as the oxygen supply system and appropriate oxygen flow rates for nasal cannulas and masks, there were notable gaps in understanding specific details. For instance, a significant portion was not well-versed in the limitations of oxygen humidification bottles, with nearly a quarter being unaware that these bottles cannot effectively humidify oxygen. Moreover, there are different understandings between Eastern and Western countries regarding the necessity of humidification during oxygen therapy. To address these gaps, targeted educational programs and related studies could be implemented. For example, integrating detailed workshops and hands-on training sessions into existing professional development curricula could improve understanding. Leveraging platforms for microlearning modules and interactive quizzes could further reinforce knowledge in an engaging manner (32, 33).

The attitude dimension showed generally positive sentiments toward the importance of education, training, and the use of humidification in low-flow oxygen therapy. However, concerns about workload increase were evident, with 63.8% of nurses agreeing or strongly agreeing that replacing humidification bottles increases workload. This perception of increased workload is consistent with findings from other nursing studies, where the adoption of new equipment or processes was associated with apprehensions about time management and additional tasks (34). To mitigate these concerns, strategies such as streamlining the humidification equipment replacement process and providing adequate staffing could be considered. Additionally, recognizing and rewarding nurses’ efforts in maintaining humidification practices through institutional acknowledgment or incentives could further enhance positive attitudes. Engaging nurses in feedback and decision-making processes about equipment use via online forums or surveys could also improve their investment in these practices (35, 36).

The practice dimension highlighted a strong adherence to best practices in monitoring and adjusting low-flow oxygen therapy, though some areas still require improvement. For example, a notable minority did not consistently educate patients on the correct use and maintenance of humidifiers, nor did they always decide the oxygen flow rate during care. This tendency to defer decision-making on oxygen flow rates may reflect hierarchical dynamics in clinical settings, a pattern seen in other studies where nurses reported limited autonomy in adjusting treatment parameters (37). To address these issues, comprehensive training sessions focusing on the importance of patient education and involvement in care decisions are recommended. Utilizing digital tools to share best practices and practical tips among healthcare professionals could standardize and improve practice. Additionally, implementing regular audits and feedback sessions can ensure adherence to protocols and highlight areas for further improvement (38, 39). This suggests that while nurses are aware of the importance of patient education, barriers such as time constraints or perceived complexity may limit its implementation, as seen in similar clinical studies (40).

It is essential to implement comprehensive training programs and continuous professional development initiatives to enhance nurses’ knowledge and practices related to low-flow oxygen therapy. To address knowledge and practice gaps, implementing simulation-based training and case-based learning could be effective approaches. Simulation-based training, which provides realistic patient care scenarios, has demonstrated significant improvements in clinical skills and decision-making among healthcare providers in intensive care and emergency departments (41). Similarly, case-based learning, involving real-life scenarios and patient cases, has proven effective in enhancing nurses’ critical thinking and practical skills in chronic disease management settings (42). Additionally, e-learning modules, which include interactive quizzes and step-by-step procedures, have been successfully used to improve healthcare workers’ knowledge and adherence to treatment protocols in infectious disease management (43).

This study has some limitations: the use of self-reported questionnaires may introduce response bias, the cross-sectional design limits the ability to infer causality, and the sample was drawn from a single hospital, which may affect the generalizability of the findings. Additionally, the exclusion of a substantial number of participants due to the strict exclusion criteria—trap questions or completion times under 60 s—could introduce selection bias. Moreover, the limited number of male nurses in the current study may influence the representativeness of gender differences in the results. However, it is worth noting that more male nurses have recently joined the nursing workforce, and we plan to conduct a multi-center study to include a greater number of male nurses in the future. Regarding the reviewer’s suggestion to assess the impact of varying levels of nurses’ knowledge and understanding on patients’ clinical outcomes, we acknowledge that this would require a longitudinal study design. The current cross-sectional study primarily aims to describe nurses’ knowledge, attitudes, and practices related to low-flow oxygen therapy. We intend to develop an interventional study design in the future, potentially incorporating longer observation periods to evaluate the effects of training interventions on both nurse competencies and patient outcomes. In addition, while this study recommends training interventions to address gaps in knowledge and practice, the specific type of intervention training is still under preparation. We are considering the use of quasi-experiment designs for future intervention research, which could help enhance training effectiveness and inform more detailed recommendations. Despite these limitations, this study provides valuable insights into the current state of nurses’ knowledge, attitudes, and practices regarding low-flow oxygen therapy and highlights the need for targeted educational interventions.

In conclusion, nurses demonstrated inadequate knowledge, positive attitudes, and inactive practices toward low-flow oxygen therapy and humidification. To improve the clinical application of low-flow oxygen therapy, it is recommended that comprehensive training programs and ongoing professional development be implemented, emphasizing both theoretical knowledge and practical skills. Enhancing these aspects can ensure more effective and consistent use of low-flow oxygen therapy, ultimately improving patient care and outcomes.

## Data Availability

The original contributions presented in the study are included in the article/Supplementary material, further inquiries can be directed to the corresponding authors.
