# Supplementary material for: Nurses’ knowledge, attitude, and practice of low-flow oxygen therapy and humidification
Source: Front Med (Lausanne). 2024 Nov 18;11:1460079. doi: 10.3389/fmed.2024.1460079 (PMC11608967; doi:10.3389/fmed.2024.1460079)
Supplement: Supplementary file 1 [file Table_1.DOCX]

**Table S1. Model fit**

| **Model** | **Ref.** | **Measured results** |
| --- | --- | --- |
| CMIN/DF | 1-3 excellent，3-5 good | 1.038 |
| RMSEA | <0.08 good | 0.012 |
| IFI | >0.8 good | 0.999 |
| TLI | >0.8 good | 0.999 |
| CFI | >0.8 good | 1.000 |
