# Supplementary material for: Nurses’ knowledge, attitude, and practice of low-flow oxygen therapy and humidification
Source: Front Med (Lausanne). 2024 Nov 18;11:1460079. doi: 10.3389/fmed.2024.1460079 (PMC11608967; doi:10.3389/fmed.2024.1460079)
Supplement: Supplementary file 2 [file Table_2.DOCX]

**Table S2 Each path coefficient**

| **Path** | | | **Estimate** | **S.E.** | **C.R.** | **P** |
| --- | --- | --- | --- | --- | --- | --- |
| Knowledge | <--- | Trained or not | 3.210 | .543 | 5.916 | *** |
| Knowledge | <--- | Dealed or not | 2.044 | .668 | 3.061 | .002 |
| Attitude | <--- | Knowledge | .379 | .044 | 8.629 | *** |
| Attitude | <--- | Gender | -1.642 | .785 | -2.091 | .037 |
| Practice | <--- | Knowledge | .395 | .063 | 6.230 | *** |
| Practice | <--- | Attitude | .340 | .077 | 4.415 | *** |
| Practice | <--- | Used or not | 1.098 | .317 | 3.466 | *** |
| Practice | <--- | Dealed or not | -1.290 | 1.244 | -1.037 | 0.300 |

*** means P<0.001
